# Sodium Acetate Responses in *Saccharomyces cerevisiae* and the Ubiquitin Ligase Rsp5

**DOI:** 10.3389/fmicb.2018.02495

**Published:** 2018-10-16

**Authors:** Akaraphol Watcharawipas, Daisuke Watanabe, Hiroshi Takagi

**Affiliations:** Division of Biological Science, Graduate School of Science and Technology, Nara Institute of Science and Technology, Nara, Japan

**Keywords:** *Saccharomyces cerevisiae*, ubiquitin ligase, Rsp5, sodium acetate responses, P-type ATPase sodium pumps, Ena1/2/5

## Abstract

Recent studies have revealed the feasibility of sodium acetate as a potentially novel inhibitor/stressor relevant to the fermentation from neutralized lignocellulosic hydrolysates. This mini-review focuses on the toxicity of sodium acetate, which is composed of both sodium and acetate ions, and on the involved cellular responses that it elicits, particularly via the high-osmolarity glycerol (HOG) pathway, the Rim101 pathway, the P-type ATPase sodium pumps Ena1/2/5, and the ubiquitin ligase Rsp5 with its adaptors. Increased understanding of cellular responses to sodium acetate would improve our understanding of how cells respond not only to different stimuli but also to composite stresses induced by multiple components (e.g., sodium and acetate) simultaneously. Moreover, unraveling the characteristics of specific stresses under industrially related conditions and the cellular responses evoked by these stresses would be a key factor in the industrial yeast strain engineering toward the increased productivity of not only bioethanol but also advanced biofuels and valuable chemicals that will be in demand in the coming era of bio-based industry.

## Introduction

The budding yeast *Saccharomyces cerevisiae* is an important microorganism for the production of alcoholic beverages, bread, and bioethanol, as well as other biochemicals due to its well-known ability during the fermentation process. *S. cerevisiae* cells possess relatively high ethanol productivity, and strong gassing power required for making dough, as well as produce distinct flavor for alcoholic beverages and bakery products (Shima and Takagi, 2009; Sasano et al., 2012a; Shiroma et al., 2014; Arshad et al., 2017). They also have lower nutrient requirement for growth and higher acid tolerance than lactic acid bacteria, which make them potentially useful for lactic acid production (Sugiyama et al., 2014). In the last decades, there has been increased interest in using *S. cerevisiae* for the production of other high value-added chemicals, e.g., isobutanol, branch-chain alcohols, amino acids, β-glucan, and lactic acids (Baek et al., 2017; Generoso et al., 2017; Mongkontanawat et al., 2018; Takpho et al., 2018). To meet these demands, researchers have considered the feasibility of using yeast cells in the presence of numerous stress conditions, e.g., weak acids, freeze-thaw, high sugar contents, oxidative treatment, and high temperature (Nakagawa et al., 2013; Sugiyama et al., 2014; Kitichantaropas et al., 2016), as well as several growth and/or fermentation inhibitors derived from feedstock biomass (Sasano et al., 2012b; Ishida et al., 2017; Jayakody et al., 2018). Thus, understanding the cellular responses of yeast in adaptation to these harsh conditions will be a key to improving yeast strains for future industrial applications.

Second-generation production of fuels and chemicals e.g., bioethanol involves the utilization of lignocellulosic biomasses such as rice straw, wheat straw, bagasse, corn fiber, and corn stover as a feedstock. These materials are comprised of 40–50% cellulose, 20–30% hemicellulose, and 10–25% lignin (Anwar et al., 2014). To release sugars (monosaccharides/disaccharides) from these biomasses, several hydrolytic processes with acid/base or enzyme are employed (Limayem and Ricke, 2012). However, not only sugars, but also growth/fermentation inhibitors including furfural, 5-hydroxymethylfurfural, vanillin, glycolaldehyde, and acetate are generated (Iwaki et al., 2013; Jonsson and Martin, 2016; Jayakody et al., 2017). In contrast to other inhibitors that can be reduced by the optimization of hydrolytic processes, acetate released from highly acetylated hemicellulose tentatively exists in lignocellulosic hydrolylates over 10 g/L at pH 5-6 (Palmqvist and Hahn-Hagerdal, 2000; Klinke et al., 2004; Almeida et al., 2007). Many studies have shown that acetate exerts an inhibitory effect on the growth and fermentation ability of *S. cerevisiae* cells (Pampulha and Loureiro-Dias, 1989; Larsson et al., 1999; Bellissimi et al., 2009). In addition, recent studies have demonstrated that acetate in the presence of sodium exerts higher growth inhibition than that in the presence of potassium (Pena et al., 2013), and sodium acetate exhibits higher cellular toxicity than sodium chloride at equal molar concentration, suggesting a synergistic inhibitory role of sodium and acetate (Watcharawipas et al., 2017). In terms of application, these findings underscore the importance of sodium acetate stress in the growth and fermentation from neutralized lignocellulosic hydrolysates.

## Sodium and Acetate Stresses: Toxicity and Adaptive Mechanisms for Yeast Cells

Acetic acid is a weak organic acid with low lipophilicity (p*K*a = 4.75). It can enter yeast cells either by passive diffusion across the plasma membrane or facilitated diffusion via the aquaglyceroporin channel Fps1 (Mollapour and Piper, 2007). At the cytosolic pH, acetic acid dissociates to acetate anions and protons in the cytoplasm, causing intracellular acidification and growth inhibition due to the perturbation of cytosolic pH homeostasis, which affects several cellular activities including signal transduction, metabolic functions, protein interaction, and cell division (Dechant et al., 2010; Young et al., 2010; Orij et al., 2012; Ullah et al., 2012; Stratford et al., 2013; Fernandez-Nino et al., 2015). Therefore, removing excess protons from the cells by the plasma membrane H^+^-ATPase Pma1 and collecting protons inside the vacuole by the vacuolar proton pump V-ATPase are suggested to be necessary for normal cytosolic pH maintenance and cell growth recovery under acetic acid stress conditions (Martinez-Munoz and Kane, 2008; Ullah et al., 2012; Stratford et al., 2013; Smardon and Kane, 2014). Moreover, the released acetate anions also negatively affect yeast cells by increasing the internal turgor pressure that leads to cell growth inhibition (Mollapour et al., 2008). In addition, the depletion of intracellular ATP pools is postulated to occur as a result of the ATP utilization by the plasma membrane H^+^-ATPase Pma1 and the vacuolar proton pump V-ATPase to pump protons out of the cells and into the vacuole, respectively (Ullah et al., 2013). Acetic acid also negatively affects the uptake of some nutrients, including glucose, tryptophan, histidine, lysine, leucine, uracil, and phosphate, which is possibly caused by either the reduction of intracellular ATP required for mediating the nutrient uptake or the downregulation of the involving genes such as *HXT1*, *HXT3*, *BAP2*, and *GAP1* genes (Kawahata et al., 2006; Ding et al., 2013). Moreover, programmed cell death was also triggered by high concentrations of acetic acid (Ludovico et al., 2002).

To cope with these cellular toxicities from acetic acid stress, *S. cerevisiae* utilizes the high-osmolarity glycerol (HOG) pathway to transduce acetic acid responses (Mollapour and Piper, 2006). The Hog1 mitogen-activated protein kinase (MAPK) phosphorylates Fps1, which triggers its ubiquitination, endocytosis, and degradation in the vacuole, thereby rendering yeast cells resistant to acetic acid (Mollapour and Piper, 2007). In addition to Hog1, the acetic acid-responsive transcriptional activator Haa1 also plays a pivotal role in acetic acid responses (Mira et al., 2011). Haa1 functions by regulating the transcription of various genes via the Haa1-responsive element (HRE) in their promoter regions (Mira et al., 2011). These genes belong to the so-called Haa1 regulon, and include *TPO2*, *TPO3*, *SAP30*, *HRK1*, *SPI1*, and *YGP1*. The drug:H^+^ antiporters Tpo2 and Tpo3 are reported to play an important role in intracellular acetate anion extrusion (Fernandes et al., 2005). It has also been suggested that the component of Rpd3L histone deacetylase complex Sap30 and the protein kinase Hrk1 are crucial for decreasing intracellular acetate contents (Mira et al., 2010). The cell wall proteins Spi1 and Ygp1 have been suggested to replenish the yeast cell wall to prevent the re-entry of acetic acid by direct diffusion (Fernandes et al., 2005; Simoes et al., 2006; Mira et al., 2011). In addition, it was shown that both laboratory and industrial strains of *S. cerevisiae* constitutively expressing *HAA1* exhibited significantly improved cell growth and initial fermentation rates under acetic acid stress (Tanaka et al., 2012; Inaba et al., 2013). Therefore, molecular breeding of industrial yeast strains lacking *FPS1* or overexpressing *HAA1* could be regarded as a promising strategy for improving acetic acid tolerance in yeast cells.

On the other hand, the pH of lignocellulosic hydrolysates after pretreatment can be increased up to the range of 5 to 6 due to neutralization (Guo and Olsson, 2014; Wilkinson et al., 2016; Bazoti et al., 2017). Under this pH condition, which is higher than the p*K*a of acetic acid (4.75), acetic acid molecules are largely present as acetate anions with lower toxicity (Mollapour et al., 2009). However, the counter ions of acetic acid (e.g., sodium) play an important role in the toxicity of acetate at higher pH (Pena et al., 2013; Watcharawipas et al., 2017). Sodium ions inhibit the growth of yeast cells via two phenomena: (i) a high concentration of sodium causes a hyperosmotic environment that induces the loss of cytoplasmic water from yeast cells (Hohmann, 2002); (ii) a high concentration of sodium increases intracellular sodium and decreases intracellular potassium contents, interfering proper cation homeostasis in yeast cells (Arino et al., 2010). At high concentrations, sodium enters the cells mainly by displacing potassium through transporters that include: (i) the high-affinity potassium transport Trk1/Trk2 system (Haro and Rodriguez-Navarro, 2002); (ii) the non-specific cation transport system named NSC1 for non-specific cation channels (Gomez et al., 1996); and (iii) the sodium-dependent phosphate transport Pi-Na ^+^ symporter Pho89 (Martinez and Persson, 1998; Serrano et al., 2002). High cytoplasmic sodium levels have been shown to negatively affect the 3′,5′-bisphosphate nucleotidase gene *HAL2* (Murguia et al., 1996), whereas proper intracellular potassium concentrations have been suggested to be necessary for several enzyme functions (Lapathitis and Kotyk, 1998).

The primary way that yeast cells cope with sodium stress is by maintaining intracellular contents and osmolarity. Hog1 phosphorylates the basic leucine-zipper transcriptional factor Sko1, leading to the upregulation of a subset of defensive genes that include the stress-inducible methylglyoxal reductase gene *GRE2*, the antioxidant peroxyredoxin gene *AHP1*, and the sodium pump gene *ENA1* (Proft and Serrano, 1999; Proft and Struhl, 2002). Ion homeostasis also involves the post-translational regulation of the sodium/proton antiporter Nha1 and the potassium channel Tok1 mediated by Hog1 phosphorylation (Proft and Struhl, 2004). To maintain suitable cytoplasmic sodium levels, surplus amounts of sodium must either be extruded through the plasma membrane by active transport via the sodium/proton antiporter Nha1 and the P-type ATPase sodium pumps Ena system or sequestered in the vacuole by the activity of Nhx1 and Vnx1, the two sodium/proton antiporters located in endosomal and vacuolar membranes, respectively (Nass et al., 1997; Cagnac et al., 2007). Additionally, the basal task of Hog1 is to increase the accumulation of glycerol as a compatible solute in response to hyperosmotic stress induced by high sodium concentration through: (i) the upregulation of glycerol biosynthesis genes *GPD1*, *GPP1*, and *GPP2* as well as the active glycerol uptake system Stl1 (Rep et al., 2000; Ferreira et al., 2005; Petelenz-Kurdziel et al., 2013); (ii) an increase of glycolytic enzyme phosphofructo-2-kinase activity (Dihazi et al., 2004); (iii) the limiting of the aquaglyceroporin channel Fps1 activity that exports glycerol (Lee et al., 2013). Besides HOG signaling, the alkaline pH-sensing Rim101 pathway also plays a role in intracellular sodium homeostasis. At alkaline pH after being C-terminally processed, the alkaline pH transcription factor Rim101 enters the nucleus to control the transcription of alkaline-responsive genes such as *ENA1* (Lamb et al., 2001; Serrano et al., 2002). In general, Rim101 and Hog1 act as the independent pathways to regulate the transcription of *ENA1.* Interestingly, a recent study showed that although Hog1, not Rim101, is predominantly required for controlling the transcription of *ENA1* under sodium chloride stress conditions, Rim101 is indispensable and has a potentially novel role in the post-translational regulation of Ena1 trafficking to the plasma membrane (Marques et al., 2015). However, detailed molecular mechanisms need to be further elucidated. In addition, previous studies also showed that Rim101 is required for tolerance to propionic acid stress due to its involvement in the transcriptional responses of *KNH1*, which encodes a protein that functions in the synthesis of cell wall β-1,6-glucan; *CWP1*, which encodes a mannoprotein that links to the β-1,3- and β-1,6-glucan in the cell wall; *BAG7*, which encodes a Rho GTPase-activating protein that plays a role in the synthesis of β-1,3-glucan by stimulating Rho1; and *YIL029c*, which encodes a protein with unknown function (Mira et al., 2009). Yeast cells lacking *RIM101* also exhibit impaired vacuole acidification, leading to acidic cytosolic pH under propionic acid stress (Mira et al., 2009). Taken together, these findings suggested that both the HOG and the Rim101 pathways potentially participate in the cellular responses to composite stress from a salt and weak acid — in this case, sodium and acetate.

## The E3 Ubiquitin Ligase Rsp5 and its Adaptors

Rsp5 (Reverses Spt^-^ phenotype protein 5) is the sole orthologue of the human Nedd4 E3 ubiquitin ligases in *S. cerevisiae*, and plays important roles in regulating physiological processes in cells — including intracellular trafficking, signal transduction, and quality control of the plasma membrane and cytosolic proteins (Dunn and Hicke, 2001; Hiraishi et al., 2009; Jarmoszewicz et al., 2012; Shiga et al., 2014) — through the interaction and ubiquitination of diverse substrate proteins. Rsp5 is composed of the N-terminal calcium-dependent phospholipid membrane binding (C2) domain, three substrate-recognizing WW domains (commonly referred to two conserved tryptophan residues in the domains), and the C-terminal catalytic ubiquitin ligase (HECT) domain (Rotin and Kumar, 2009).

The essential role of Rsp5 is attributed to its activity in the regulation of the *OLE1* gene expression via ubiquitination-mediated proteolytic processing of the transcriptional activators Spt23 and Mga2 that localize at the endoplasmic reticulum (ER) (Zhang et al., 1999; Hoppe et al., 2000; Shcherbik et al., 2003, 2004). Rsp5 is known to downregulate various plasma membrane transporters for nutrients and ions as well as receptors. This downregulation contributes both to protein quality control mechanisms in which the plasma membrane proteins are misfolded (e.g., in response to heat stress) and regulatory mechanisms in which the transporters are endocytosed to restrict their activity or receptors are degraded for their desensitization. For example, the ubiquitination by Rsp5 mediates the endocytosis of the general amino acid permease Gap1 in response to the shifting from a poor nitrogen source to ammonium ions (Springael et al., 1999) and in response to environmental stresses such as ethanol, hydrogen peroxide, high temperature, and lithium chloride (Hoshikawa et al., 2003; Shiga et al., 2014). Our previous studies also suggest that Rsp5 participates in the maintenance of stress-induced abnormal proteins through degradation or repair process (Haitani et al., 2006; Haitani and Takagi, 2008; Hiraishi et al., 2009). It has been shown that Gap1 remains stable on the plasma membrane under ethanol stress in the stress-hypersensitive *rsp5*^A401E^ mutant (Hoshikawa et al., 2003; Shiga et al., 2014). On the other hand, constitutive inactivation and endocytosis of Gap1 was effectively mediated by the *rsp5*^T357A/K764E^ mutant (Haitani et al., 2009). Interestingly, a novel mechanism, in which Rsp5 is dephosphorylated and activated when a rich nitrogen source is supplied and vice versa, was also proposed to involve the regulation of Gap1 ubiquitination (Sasaki and Takagi, 2013).

Moreover, the endocytosis of the maltose permease Mal61, the hexose transporter Hxt6/7, the uracil permease Fur4, the tryptophan permease Tat2, the zinc transporter Zrt1, and the divalent cation transporter Smf1 is regulated via the ubiquitination by Rsp5 in response to various stimuli (Galan et al., 1996; Hein and Andre, 1997; Krampe et al., 1998; Medintz et al., 1998; Beck et al., 1999; Gitan and Eide, 2000; Sullivan et al., 2007; Nikko and Pelham, 2009). The internalization of the pheromone receptor Ste2 after pheromone sensing is also controlled by the ubiquitination mediated by Rsp5 as a part of its desensitization (Dunn and Hicke, 2001). In addition, Rsp5 ubiquitin ligase activity has been shown to be important for fluid phase endocytosis (Dunn and Hicke, 2001). Recently, the plasma membrane H^+^-ATPase Pma1 was shown to be mono-ubiquitinated by Rsp5, leading to its internalization and vacuolar degradation, in response to the loss of V-ATPase activity (Smardon and Kane, 2014), suggesting that Rsp5 plays a role in the regulation of cytosolic pH homeostasis in yeast cells. In summary, Rsp5 plays important physiological roles in various cellular processes. However, our knowledge of the role of Rsp5 in regulation of the monovalent cation transporters Ena1/2/5 is still limited.

To catalyze the ubiquitination on a particular substrate, Rsp5 has to interact with the substrate through the interaction between the WW domains of Rsp5 and the PY motifs consisting of short peptide sequences (XPXY) located in the substrate. However, most of the Rsp5 substrates do not contain the PY motifs (Gupta et al., 2007), suggesting the requirement of PY-motifs containing adaptor proteins. The evidence supporting the existence of the Rsp5 adaptors originates from the identification of Bsd2 and Tre1/2, which contain the PY motifs, as the proteins required for the ubiquitination of the divalent cation transporter Smf1 (Liu et al., 1997; Hettema et al., 2004; Stimpson et al., 2006). In addition, Bsd2, but not Tre1/2, is found to be crucial for ubiquitination and trafficking of the precursor of the vacuolar enzyme polyphosphatase Phm5 (Hettema et al., 2004). This suggests that combination of adaptors can also affect the substrate specificity by Rsp5 (Watanabe et al., 2015). In addition, many Rsp5 adaptor proteins have been identified, including Bul1/2 (Soetens et al., 2001; Liu et al., 2007), Ear1 and its homologue Ssh4 (Leon et al., 2008), as well as the arrestin-related trafficking adaptor (ART) protein family members, which consist of Art1 to Art9 (Lin et al., 2008; **Table 1**).

**Table 1:** Lists of Rsp5 adaptor proteins, conserved domains, and their locations.

Adaptor protein	Conserved domain	Location
Bsd2	TM	Golgi-endosome
Tre1/2	TM, TFR dimer, PA	Golgi-endosome
Ear1/Ssh4	TM, B30.2/SPRY	Golgi-endosome
Bul1/2	Bul1 N-terminus, Bul1 C-terminus	Plasma membrane, Golgi-endosome
Bul3	Bul1 N-terminus	-ND-
Art1	Arrestin N-terminus	Plasma membrane
Art2	Arrestin C-terminus	Plasma membrane
Art3	Arrestin N-terminus, Arrestin C-terminus	-ND-
Art4	Arrestin N-terminus, Arrestin C-terminus	Plasma membrane
Art5	Arrestin C-terminus	Plasma membrane
Art6	Arrestin C-terminus	-ND-
Art7	Arrestin N-terminus, Arrestin C-terminus	-ND-
Art8	Arrestin C-terminus	Plasma membrane
Art9 (Rim8)	Arrestin N-terminus, Arrestin C-terminus	Plasma membrane
Art10	-ND-	-ND-


*TM, Predicted transmembrane domain; TFR dimer, Transferrin receptor-like dimerization domain; PA, protease-associated domain; B30.2/SPRY, B30.2 and/or SP1a/RYanodine receptor domain; and ND, Not determined (Information was obtained from *Saccharomyces* Genome Database (SGD) and Lauwers et al., 2010).*

## Prospective Roles of Rsp5 and its Adaptors in Sodium Acetate Responses

We can summarize our understanding of the cellular responses to sodium acetate as follows. Previous studies have shown that acetate stress in the presence of sodium at pH 6.8 exhibits a growth-inhibitory effect and triggers Hog1 MAPK phosphorylation, which leads to upregulation of the *GPD1* mRNA level and thereby increased accumulation of intracellular glycerol (Mollapour and Piper, 2006; Mollapour et al., 2009). Our study also found that disruption of *HOG1* conferred sodium acetate sensitivity on yeast cells, but did not significantly affect the accumulation of intracellular sodium in yeast cells under sodium acetate stress conditions, suggesting that Hog1 mediated sodium acetate responses via other components e.g., glycerol (Watcharawipas et al., 2017). Thus, these studies indicate that one of the sodium acetate responses at higher pH is to increase intracellular osmolarity via the accumulation of glycerol, which allows the cells to counteract the loss of cytoplasmic water. Our recent study also revealed that the full acquisition of tolerance to sodium acetate is essentially dependent on the Rim101 pathway, since the disruption of *RIM8*, *RIM20*, and *RIM101* causes sodium acetate sensitivity on yeast cells (Watcharawipas et al., 2017). Moreover, we found that yeast cells lacking *RIM8* showed an increased accumulation of intracellular sodium content under sodium acetate stress conditions (Watcharawipas et al., 2017) similar to that observed under sodium chloride stress conditions (Marques et al., 2015), supporting the finding that the Rim101 pathway is crucial for the proper transport and accumulation of Ena1 on the plasma membrane (Marques et al., 2015). However, whether the Rim101 pathway has a principal role in regulating the transcription of *ENA1* in the presence of sodium acetate still needs to be clarified. This might also explain why the disruption of *HOG1* did not affect the intracellular sodium level under sodium acetate stress conditions. Thus, these results suggest that the HOG signaling and the Rim101 pathway independently play important roles in the sodium acetate responses in *S. cerevisiae* cells. Further investigation showed that the triple disruption of *ENA1/2/5*, which is downstream of the HOG and Rim101 pathways, confers sodium acetate sensitivity and increases the intracellular sodium accumulation in yeast cells under sodium acetate stress (Watcharawipas et al., 2017). Previous studies also show that the copy number of P-type ATPase sodium extrusion pump *ENA1/2/5* genes is associated with acetate tolerance in the presence of sodium (Gilbert et al., 2009; Pena et al., 2013). Another study shows that the sodium/proton antiporters Nha1, Nhx1, and Vnx1 located at the plasma membrane, endosome, and vacuole, respectively, are involved in the cellular adaptation under sodium acetate stress at initial growth phase (Yang et al., 2010). Thus, these studies underscore the great importance of the Ena1/2/5 sodium pumps for the realization of sodium acetate tolerance, and imply that, at the very least, the sodium acetate responses involve the extrusion of sodium out of the cells or the sequestration of sodium inside the vacuole in the presence of acetate. Importantly, the intracellular sodium accumulation in the presence of sodium and acetate is higher than that in the presence of sodium and chloride, indicating the synergistic inhibitory effect of acetate anions (Watcharawipas et al., 2017). It would be intriguing to further investigate whether the acetate anions negatively impact the Ena1/2/5 activity.

**FIGURE 1:**
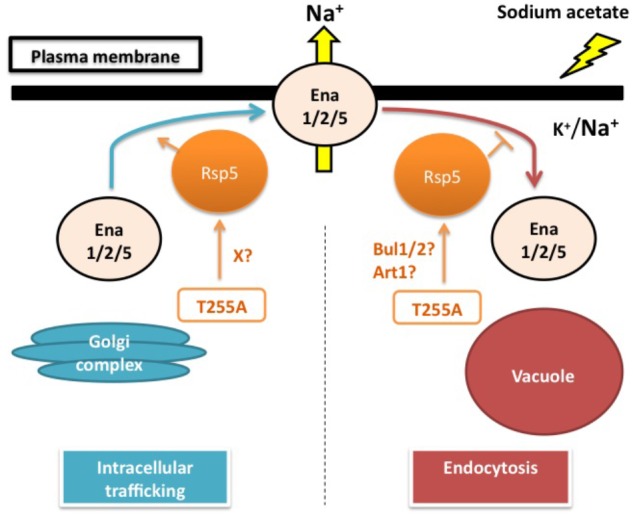
Sodium acetate responses by yeast cells: hypothesized role of Rsp5 in regulating the sodium pumps Ena1/2/5. Sodium acetate triggers the increased accumulation of intracellular sodium. In this model, Rsp5 is hypothesized to play a post-translational regulatory role in either intracellular trafficking of Ena1/2/5 from the Golgi complex to the plasma membrane or endocytosis of Ena1/2/5 from the plasma membrane to the vacuole. The T255A mutation might either promote the intracellular trafficking of Ena1/2/5 through an unidentified factor (X) or inhibit the endocytosis of Ena1/2/5 via Bul1/2 or Art1 adaptor proteins, thereby enhancing the sodium extruding activity of Ena1/2/5 on the plasma membrane under sodium acetate stress conditions.

The physiological importance of Rsp5 in the sodium acetate responses has been suggested by the sodium acetate sensitivity of *rsp5*^L733S^ (*rsp5-1*) and *rsp5*^A401E^, which have defects in the catalytic HECT domain and the WW3 domain, respectively (Watcharawipas et al., 2017). Recently, Wijayanti et al. (2015) found that Thr255Ala, an amino acid substitution in the WW1 domain, renders yeast cells the tolerance to sodium acetate with higher initial growth rate. Intriguingly, the changed threonine residue (Thr255) is conserved among the WW domains of Nedd4-family ubiquitin ligases and has corresponding positions at Thr357 in WW2 and Thr413 in WW3 of Rsp5. These threonine residues are also the putative phosphorylation sites which may play an important role in the substrate specificity of Rsp5 (Sasaki and Takagi, 2013; Watanabe et al., 2015). Interestingly, the Thr255Ala variant causes lower intracellular sodium accumulation than wild-type cells under sodium acetate stress. This sodium level difference is canceled by the triple deletion of *ENA1/2/5*, suggesting that the *rsp5*^T255A^ mutant positively affects the sodium extruding activity through Ena1/2/5. Generally, Rsp5 post-translationally controls several plasma membrane proteins under various conditions. Thus, it is possible that Rsp5 might affect either Ena1/2/5 trafficking to the plasma membrane or Ena1/2/5 endocytosis. However, Ena1/2/5 does not have the PY motifs. For this reason, it is hypothesized that adaptor proteins are required for mediating the regulation of Ena1/2/5 by Rsp5. The Rsp5 adaptors Rim8, Bul1/2 and Art1 have been shown to be important for the sodium acetate tolerance in yeast cells. However, the Rsp5-Rim8 interaction and Rim8 mono-ubiquitination by Rsp5 are dispensable for sodium acetate tolerance (Watcharawipas et al., 2017). It would be intriguing to further examine the roles of Bul1/2 or Art1 in sodium acetate responses (**Figure 1**).

## Conclusion

In this mini-review, we discuss the current understanding of sodium acetate stress as a composite stress of sodium and acetate, which may be able to influence each other. Thus, the cellular responses involve not only individual responses to sodium or to acetate, but also integrated actions to combat the effects of both. In addition, we shed light on a potentially important link—namely, that the protein ubiquitination system mediated by the E3 ubiquitin ligase Rsp5 possesses an important role in selectively regulating intracellular sodium homeostasis under sodium acetate stress, potentially through the P-type ATPases Ena1/2/5 and Rsp5 adaptor proteins. Further investigations will uncover the evolutionarily conserved role of Nedd4-family ubiquitin ligases, and will also benefit industrial applications through an improved understanding of their related stress conditions.

## Author Contributions

AW, DW, and HT analyzed the data and drafted the manuscript. AW prepared the figure and table. HT coordinated the manuscript preparation. All authors reviewed and approved the final version of manuscript.

## Conflict of Interest Statement

The authors declare that the research was conducted in the absence of any commercial or financial relationships that could be construed as a potential conflict of interest.
